# Assessing Virtual Mental Health Access for Refugees during the COVID-19 Pandemic Using the Levesque Client-Centered Framework: What Have We Learned and How Will We Plan for the Future?

**DOI:** 10.3390/ijerph19095001

**Published:** 2022-04-20

**Authors:** Michaela Hynie, Annie Jaimes, Anna Oda, Marjolaine Rivest-Beauregard, Laura Perez Gonzalez, Nicole Ives, Farah Ahmad, Ben C. H. Kuo, Neil Arya, Nimo Bokore, Kwame McKenzie

**Affiliations:** 1 Department of Psychology, York University, Toronto, ON M3J 1P3, Canada; 2 Center for Refugee Studies, York University, Toronto, ON M3J 1P3, Canada; annaoda@yorku.ca (A.O.); laurapg@yorku.ca (L.P.G.); 3 Department of Psychoeducation, Sherbrooke University, Sherbrooke, QC J1K 2R1, Canada; annie.jaimes@usherbrooke.ca; 4 Sherpa University Institute, Montreal, QC H3N 1Y9, Canada; nicole.ives@mcgill.ca; 5 Department of Psychiatry, McGill University, Montreal, QC H3A 1A1, Canada; marjolaine.rivest-beauregard@mail.mcgill.ca; 6 School of Social Work, McGill University, Montreal, QC H3A 1B9, Canada; 7 School of Health Policy and Management, York University, Toronto, ON M3J 1P3, Canada; farahmad@yorku.ca; 8 Department of Psychology, University of Windsor, Windsor, ON N9B 3P4, Canada; benkuo@uwindsor.ca; 9 Department of Family Medicine, McMaster University, Hamilton, ON L8S 3L8, Canada; narya@uwaterloo.ca; 10 School of Social Work, Carleton University, Ottawa, ON K1S 5B6, Canada; nimobokore@cunet.carleton.ca; 11 Wellesley Institute, Toronto, ON M5A 2E7, Canada; kwame@wellesleyinstitute.com

**Keywords:** mental health care access, refugees, Canada, telemedicine, virtual therapy, client-centered framework

## Abstract

During the COVID-19 pandemic, mental health services rapidly transitioned to virtual care. Although such services can improve access for underserved populations, they may also present unique challenges, especially for refugee newcomers. This study examined the multidimensional nature of access to virtual mental health (VMH) care for refugee newcomers during the COVID-19 pandemic, using Levesque et al.’s Client-Centered Framework for Assessing Access to Health Care. One hundred and eight structured and semi structured interviews were conducted in four Canadian provinces (8 community leaders, 37 newcomer clients, 63 mental health or service providers or managers). Deductive qualitative analysis, based on the Client-Centered Framework, identified several overarching themes: challenges due to the cost and complexity of using technology; comfort for VMH outside clinical settings; sustainability post-COVID-19; and communication and the therapeutic alliance. Mental health organizations, community organizations, and service providers can improve access to (virtual) mental health care for refugee newcomers by addressing cultural and structural barriers, tailoring services, and offering choice and flexibility to newcomers.

## 1. Introduction

In recent years, the number of refugees, asylum seekers, and internally displaced people has been growing; in 2021, 26.6 million refugees fled life threatening situations due to conflicts, wars, and acts of violent extremism [1]. Refugees across the world constitute a particularly vulnerable and underserved population. While forced migration and armed conflict appear to be associated with an elevated prevalence of PTSD, anxiety, and depression in refugee populations [2], various factors in the resettled country play an important role in increasing psychosocial vulnerability [3]. COVID-19 has starkly increased pre-existing inequalities, disproportionately affecting vulnerable groups, such as refugee newcomers. Indeed, the pandemic’s burden of infection, death, and socio-economic impacts has largely affected poorer populations, disadvantaged ethnic groups, migrants, low paid essential workers, and people lacking social protection or living in crowded housing [4,5], making refugee newcomers particularly vulnerable to the cumulative impact of multiple forms of marginalization.

Access to health and mental health care, a key social determinant of health inequalities [6], has been a challenge for refugee newcomers, and may have worsened for them during the global health crisis. However, the pandemic has also brought potential opportunities regarding access to care, including access through virtual modalities. Mental health institutions and community organizations have rapidly sought to adapt to public health measures by offering virtual services. Virtual mental health services include phone, internet-based voice or video interactions, and text-based applications or messaging. Although virtual modalities offer interesting avenues in times of confinement, there are limited data assessing if they uphold their promise of increasing access to care for disenfranchised populations, such as refugee newcomers, or even exacerbate inequities. The goal of this exploratory project was to document the perceptions of refugee newcomers, as well as those of key actors involved in the referral and delivery of virtual mental health (VMH) services, to understand how virtual modalities can impact access to mental health services for vulnerable groups.

### 1.1. Context of the Study

Canada has welcomed more than a million refugees since the 1980s through a host of different programs. In resettlement programs, refugees are selected by a host country and enter with permanent resident status [7]. In Canada, resettled refugees receive financial and settlement support for at least the first year of residency, which can facilitate access to services. During the first year, the Interim Federal Health Insurance Program (IFHP) covers supplemental health care not usually included in most provincial health plans [8], like non-physician mental health services, plus basic services until provincial coverage is obtained. Although the Canada Health Act aims to facilitate barrier-free access to health care for all residents [9], the availability and accessibility of mental health care for refugee newcomers is less than ideal. Despite facing higher risks for psychological and mental health difficulties, refugees are known to present relatively low rates of help-seeking for mental health services [10,11]. Access to mental health care can be limited by financial costs, but also cultural and structural obstacles: low income; unemployment; racial discrimination; literacy; housing; social exclusion; stigma; perceptions of health, mental health, and services; and linguistic barriers, etc. [12,13].

As elsewhere, COVID-19 has forced many Canadian health and mental health providers to offer services through virtual platforms, with some differences across provinces and fluctuations through different waves of the pandemic. This might have directly affected accessibility for vulnerable populations, but also indirectly, by making referral to services more challenging for their service and health providers. Settlement workers, case managers, and primary health care providers are the main points of contact between refugees and mental health and social services. These providers’ ability to assess refugee newcomers’ needs and capacities, and the accessibility of available services, are essential in connecting refugee newcomers to available care [14].

### 1.2. Conceptualizing the Role of Virtual Mental Health Services in Promoting Access and Improving Service Disparity

Even prior to the COVID-19 pandemic, with the rapid advancement of digital technologies, incorporation of VMH services such as telepsychology and teletherapy into mainstream psychological practices was gaining increasing attention by mental health professionals and service providers [15,16]. Proponents of virtual care underscore the potential of these services to improve access to psychological interventions and reduce service disparity for marginalized groups, including racialized and newcomer groups [17]. Despite their promises, however, there seems to be a “research to practice gap” [18,19] in virtual health, which has been attributed to a lack of user input in natural (vs. lab) settings. Indeed, online psychological interventions have been met with some resistance, including from mental health clinicians, around perceptions of telepsychology, lack of training, concern over legal and professional regulations, and reimbursement issues, for example [20,21]. Moreover, new health interventions can initially widen health inequalities, selectively improving services only for privileged or relatively advantaged users, suggesting that the shift to virtual care may not have benefitted refugee newcomers, who face multiple barriers to access [22]. Access and implementation of VMH care thus needs to be conceptualized from the perspectives of all relevant stakeholders, including clients, therapists, the operational frameworks of organizations, the larger health systems, funders, and policy makers [23].

### 1.3. Theoretical Framework

This project is grounded in Levesque, Harris, and Russell’s [24] Client-Centered Framework for Assessing Access to Healthcare (referred to as the Client-Centered Framework from here on). The framework describes access to health care services as a function of the complex interface between the characteristics of the services, service providers, health systems, and organizations on one side, and clients/patients and their environments, on the other. To fully understand the complexities of access, Levesque and colleagues separate out five supply-side factors (approachability, acceptability, availability and accommodation, affordability, and appropriateness) and five demand-side factors (ability to perceive, ability to seek, ability to reach, ability to pay, and ability to engage) taking into account environmental contexts.

There are multiple opportunities for different trajectories depending on the health systems and the population perspectives. Adopting this framework to assess newcomers’ and service providers’ perceptions of VMH services during the COVID-19 pandemic allows for understanding how these trajectories behave in the context of system-wide regulations, impacting all stages in the health care seeking continuum for all service users in the same time period. Thus, this project aimed to better understand the accessibility of VMH care during the COVID-19 pandemic from the perspective of both refugee newcomer clients and providers offering or referring to VMH services, to support access to more equitable, effective, and appropriate VMH services for refugee newcomers across Canada.

## 2. Materials and Methods

### 2.1. Study Design and Context

This paper describes findings from the qualitative arm of a larger mixed-methods exploratory study examining refugee newcomers’ access to VMH care, conducted between November 2020 and May 2021 in Alberta, British Columbia, Ontario, and Quebec—the four Canadian provinces with the highest numbers of resettled refugee newcomers. Questions regarding providers’ perceptions of challenges in the delivery of VMH care and access to resources and training made up the mixed-methods part of the study. This paper reports on the qualitative assessment of access to VMH services using data from interviews with community leaders, health and mental health providers, managers and newcomer clients, and front-line providers. Focus groups were planned with providers, but aside from 6 small group interviews, individual interviews were utilized instead due to recruitment challenges.

The study was guided by two advisory committees. The first was composed of 11 providers and policy makers working with refugee newcomers in the four provinces, the second of 11 newcomers from the Afghan, Congolese, Eritrean, Ethiopian, Iranian, and Syrian communities in these same provinces. Advisory committee members supported participant recruitment and interpretation of findings, and co-developed interview materials and strategies for dissemination.

### 2.2. Participant Recruitment and Procedures

#### 2.2.1. Key Informants

Service providers (program coordinators, managers and directors of settlement organizations, settlement workers, health and mental health care providers, and interpreters) and community leaders were recruited through emails sent through the project’s advisory committees and the research team’s networks, and national and regional networks working with refugees across Canada. Inclusion criteria for service providers included a minimum of three years working with refugees, fluency in English or French, and engagement in either settlement or (mental) health care work. Inclusion criteria for community leaders included arriving in Canada as a refugee in the past 15 years, knowledge of mental health issues in their communities, being over 18, being able to provide consent, and being able to understand and speak English or French. No additional demographic information was collected.

#### 2.2.2. Refugee Newcomer Clients

Recruitment of refugee newcomers was conducted through group emails and public invitations sent through community groups, settlement agencies, university health services, health networks, and through snowball sampling by peer researchers, and through research team member networks, and advisory committee networks. Inclusion criteria for refugee newcomers included living in Canada for 5 years or less, being over 18, having personal or family member experience with mental health services, being able to provide consent, and being able to understand and speak Amharic, Arabic, English, Farsi, French, Somali, Spanish, or Tigrinya. Refugee newcomers were offered a small honorarium for their participation.

#### 2.2.3. Front-Line Service Provider Interviews

Front-line service providers were recruited through the same networks as the key informants plus targeted snowball sampling to fill particular categories of services (e.g., serving francophone clients, serving children and youth) or regions.

All participants provided e-mailed written consent prior to the interviews. Key informant interviews and service provider follow-up interviews lasted approximately 60 min, while refugee newcomer client interviews lasted approximately 30 min. All interviews were audio recorded, transcribed, and translated into English or French where necessary. Written notes were also taken during the interviews.

### 2.3. Data Collection

The participation of service providers and refugee newcomers involved phone or online interviews in the language of their choice, where possible, with one or two team members. Interview grids for each category of actors (newcomer versus provider) were derived from the Client-Centered Framework [24], modified to probe elements of virtual access, and thus addressed similar themes but emphasized different stakeholder experiences.

Semi-structured key informant provider interviews were broader and included more probes and questions around the impact of COVID-19 on newcomer mental health and the broader context of virtual care in their agency and/or profession. Semi-structured follow-up provider interviews addressed new issues emerging from key informant interviews. They included a brief professional history and focused on front-line provision of services or referrals to elicit more focused information on barriers and facilitators to access, and training and support for providers (the latter is reported elsewhere). No other demographic information was collected.

Semi-structured interviews with community leader key informants explored community mental health experiences and issues prior to and during the COVID-19 pandemic, as well as determinants of needs, and access and accessibility factors. Again, no other demographic information was collected.

Refugee newcomer clients answered a short demographic questionnaire during the interview. Interview questions were structured and assessed determinants of needs, access, and accessibility as identified by the key informant interviews, with focused probes relevant to technology access, literacy, satisfaction, and preferences. Interview questions were translated by a professional translator into Amharic, Arabic, English, Farsi, French, Somali, Spanish, or Tigrinya, and linguistically and culturally validated by the project’s peer researchers.

Ethics approval was granted by the Institutional Review Boards of three institutions.

### 2.4. Qualitative Analysis

Data analysis followed Thomas and Harden’s [25] thematic analysis stages but with some modifications to manage the large amount of data. Six team members collaborated to develop a codebook for deductive analysis based on the Client-Centered Framework, plus codes generated inductively through the reading of the transcripts. Three coders conducted holistic coding of all transcripts. Detailed coding was conducted within the holistic codes by a fourth team member, who then organized the detailed codes into ‘descriptive’ themes. ‘Analytical’ themes were decided by assessing the richness, breadth, and depth of descriptive themes, significance, and commonality among interviews, and fit with the conceptual framework. The process was iterative and multiple meetings were scheduled during each phase. Validity was addressed by checking and confirming coding and interpretations with the full coding team in each phase, and by actively searching for disconfirming evidence in the data.

Given the heterogeneity of VMH services offered/experienced by participants across provinces, organizations, and individuals, the perspectives of newcomers and providers are described together mainly as complementary perspectives, enriching our emerging understanding of the phenomena.

## 3. Results

### 3.1. Participants

This study used a convenience sample and snowball recruitment strategies across four provinces. Across 108 key informant and follow-up interviews, there were 45 representatives of refugee communities (Alberta, *n* = 9; BC, *n* = 5; Ontario, *n* = 26; Quebec, *n* = 5) and 63 providers and managers (Alberta, *n* = 4; BC, *n* = 10; Ontario, *n* = 25; Quebec, *n* = 24).

#### 3.1.1. Front-Line Provider Professional Information

Providers were asked in the interview how long they had worked with refugee populations and what proportion of their clients were refugees. We had intended to include a brief survey with the consent form for sociodemographic information including gender and age, but unfortunately, this was not sent. Professionals had worked a median of 5 years, ranging from a year to 27 years; almost a third (*n* = 19) reported more than 10 years’ experience. About 84% reported that refugees and/or asylum seekers made up more than half of their clientele. The distribution of professions by province is presented in Table 1.

#### 3.1.2. Representatives of Refugee Communities

Key informants: Key informant community leaders were residents of Ontario (*n* = 4; 50%), Québec (*n* = 2; 25%), and Alberta (*n* = 2; 25%). Community leaders in some cases saw themselves representing and describing specific ethno-cultural communities (Eritrean, *n* = 2; Ethiopian and Eritrean, *n* = 2; Syrian, *n* = 1), but in other cases saw themselves as representing broad categories of newcomers (West Asian/Arabic speaking, *n* = 1; Muslim, *n* = 2), and describing shared experiences of these broader communities.

Clients: Refugee newcomer clients ranged in age from 20 to 56 years old (*M* = 35.4, *SD* = 9.8), and had been in Canada from a few months to up to five years. More than half (*n* = 21, 58.3%) self-identified as female. The majority (*n* = 27) reported personal experience accessing mental health services, four reported a family member accessing, and six a mix of family member, own experience, and community experience. More information about refugee newcomer clients can be found in Table 2.

### 3.2. Dimensions of VMH Service Accessibility

Consistent with Levesque et al.’s [24] Client-Centered Framework, the findings are organized by the characteristics of the services and resources of the newcomer clients. Both clients and providers commented on each aspect of access. Results from the key informants and subsequent interviews, and the different participant groups, are presented together, but the participant roles are identified for each quote.

#### 3.2.1. Approachability

Approachability refers to the ease with which services can be identified and reached.

Networks: Newcomers and providers of health and social services both described how informal and formal networks played an important role in ensuring awareness of mental health services. Newcomers relied on networks of friends, sponsors, health and social providers for information about services. This highlights the importance of communities’ knowledge of existing services, but also the value of holistic services; accessing one service increased the opportunities for information and awareness of other services, including those related to mental health.

For service providers referring clients to specialized care, the challenge was identifying what services were currently available for their clients, and in what modality. Providers who relied on previous referral relationships easily continued referring their clients for specialized care. Where those relationships were less well-defined or established, providers reported resorting to arduous online searching in the altered service environment.

Online outreach and resources: Making services visible to clients and providers was more challenging in a virtual environment. Agencies used direct outreach to their existing clients, a strategy that clients noted was important for ensuring awareness of available services and acting on that awareness. This highlights the relative vulnerability of isolated newcomers who were not connected to social or settlement services. Providers noted that reaching new clients outside of their existing networks was more challenging during the pandemic and required a greater use of intentional promotion.

*...with COVID and with the isolation, many persons would probably–could have been going out to libraries or to a community center for something, I mean, [and] just by chance heard somebody talking about, “oh, you know, there’s a service, do we miss that piece?” So, no, it’s, it’s more so on organizations really trying to put a lot on perhaps their websites or on social media.*

(Mental health intake and assessment worker 1)

Websites, social media, and online events like Facebook live community discussions became the main method used by both newcomer communities and agencies to increase awareness or visibility of mental health services. Agencies in some sites also reported efforts to build and support searchable databases of available services for both providers and clients, to facilitate access to information about which services were available, who had waiting lists, and in which modality services were offered.

#### 3.2.2. Acceptability

Acceptability refers to social factors that make services acceptable (or not) for clients, with a recognition that care can be offered in ways that make it more acceptable to some members of the population than others.

Not ideal, but at least available: Both providers and newcomer clients generally saw virtual services as helpful and acceptable and they were grateful that services were at least available. However, both clients and providers suggested that VMH services are not always ideal and many preferred in-person services.

*I find virtual, somehow, even if it doesn’t cover everything we’re supposed to cover as before- but trying to do the service from home and virtually—I’m being there for them, all the time; making them happy and feeling like there’s some people behind their back, they[‘re] helping them.*

(Mental health provider key informant 1)

Which modalities are acceptable for whom: Cultural acceptability played a role in preferences for same-culture therapists and in some cases intersected with delivery modality. While VMH services were generally seen as acceptable, some methods of virtual services were deemed to be less culturally appropriate than others in certain communities, as noted by this newcomer client:

*[...] we are from an oral tradition. We talk much more than we write. So by textos or online, such as chatting with someone and e-mailing, it is not our tradition or nature. But talking with someone, like I told you, like now … At least you feel that there is someone on the other side.*

(Refugee newcomer 2)

The use of cameras, in particular, was described as making some people particularly uncomfortable, such as older adults. Discomfort with cameras may be particularly salient in group settings. Although we anticipated gendered concerns around the use of cameras, this did not emerge in our data except in the context of transgendered clients.

*I recently created support group for the LGBT community specifically for transgender female to male... Some of them, … They don’t want to appear on the camera because they belong to the same community. [...]they don’t want anybody to know that they are transgender or LGBT so I’m going to try again to do it in a different way.*

(Mental health provider key informant 2)

Providers in particular raised concerns about the acceptability of virtual modalities for people coping with serious mental illnesses or with clients who were dealing with trauma. One gendered concern that emerged was that virtual services were described as challenging in situations of domestic violence, which in this study was always raised in the context of violence against women; finding safe spaces in which to access virtual services was difficult when one shared their home with the perpetrator of the violence.

*I remember I had a woman, she used to talk when her husband was outside. They try to figure out a time, yeah. Sometimes she calls and she says a few words, and she doesn’t discuss her situation—it’s really hard, it’s not easy. *

(Mental health provider key informant 3)

Language preferences in virtual modalities: One of the greatest advantages of virtual care was increasing the ability to accommodate language needs because it was possible to connect with clinicians or interpreters outside of the immediate community.

*If they have to go in person, it could be a challenge. We have to arrange for interpretation. We have to arrange for transportation. We have, you know, but with virtual services they can even reach out to mental health therapist in [names different cities]...*

(Settlement service provider 1)

Findings around virtual interpretation were mixed. Many providers felt that it went smoothly and enhanced accessibility, and even that their clients preferred to have a virtual interpreter because they felt that their privacy was better protected. However, some newcomers and providers also reported technological barriers, such as dropped calls or discomfort, as noted by this provider:

*Again you know a lot of my staff team aren’t techie so trying to do a three-way call or getting someone outside of our organizations to join into a video has not been as efficient.*

(Agency director key informant 1)

Some providers also reported that clients could be less trusting of interpreters in the virtual space, as in the following discussion about issues of trust and its relation to past trauma:

*[...] one of the considerations for the virtual platform is that the interpreter has to be able to easily access that platform, they have to be linked in, but just over the phone. I think if it’s three anonymous people [i.e., the client, the therapist, and the interpreter]…when it’s you as the provider (that maybe they’ve not met before), the patient, and then there’s this other person who speaks their language that they can’t see... I think sometimes, it’s a little bit- it depends again. It depends on the individual or even the group. I know that we’ve had a lot of issues with our Yazidi just because they are so distrustful because of their trauma.*

(Primary health care provider 1)

Providers also noted that some interpreters had limits on the kinds of technology they would use, and this was a challenge when dealing with interpreters of less prevalent languages, where there were fewer interpreters to choose from. Interpretation services in group settings with multiple languages were also described as more challenging with virtual modalities.

#### 3.2.3. Availability and Accommodation

Availability and accommodation refer to being able to access services physically (in our case virtually), and in a timely manner.

Bridging the challenge of distance: Both newcomer clients and providers noted that clients were spared the time, cost, and inconvenience of having to travel long distances to appointments, noting also the high cost of public transportation. This may be a particular advantage for caregivers of young children. Virtual modalities also allowed for more frequent check-ins and greater flexibility for staff to accompany clients on their appointments. Thus, virtual services enhanced availability in a number of ways.

Navigating changes and negotiating accommodations: Many providers employed important service adaptations to ensure that care remained available and ideally adapted to clients’ preferences. Although some newcomers reported having no choice of modality, most providers reported being able to offer some choices to their clients, and about half also provided some in-person care, as permitted, when clients needed such services. Accommodating clients’ modality preferences was valued not just for enhancing access but also as a way to build a therapeutic relationship and validate clients.

Our findings also underline how policies at provincial and institutional levels supported different options for providers, in terms of modalities, applications and software, as well as training and support. Providers were often limited by security concerns and/or professional body standards and could find themselves caught between the preference of clients for specific modalities and expectations of their agency for specified options.

*Sometimes you just have to see somebody and it just- I don’t have a sanctioned way to access people over video conferencing. … if I have them try to connect through Microsoft Teams, which is 100% confidential but it requires so much digital literacy people can’t access it, people just can’t. So I have to say this is a huge—it’s a huge frustration for me and telephone calls work only so far.*

(Primary health care provider 1)

Flexibility of schedules: Newcomer clients also reported wanting more flexibility in terms of time of day or days of the week that services were offered. Although some providers noted that they had more flexible schedules now that they were working virtually, they generally did not identify this as a need. Rather, providers and managers noted that working from home could make it harder to maintain boundaries around staff work hours. There is a potential conflict here between refugee newcomers’ need/preference for flexible hours and providers’ need to protect work–life balance when working from home in a time of increased demands on mental health services and staff.

#### 3.2.4. Affordability

Affordability refers to the cost of appropriate services, in both material resources and time.

The cost of technology: In most Canadian provinces, non-physician mental health services for Canadian residents can involve fees, and virtual modalities can add additional costs. Both clients and providers noted challenges in terms of the cost of devices, data plans, and reliable internet services. Some providers and agency directors described financial/resource support for virtual services that helped them build and support virtual service infrastructure to respond to COVID-19 restrictions. Some agencies tried to ensure access to devices and sometimes also subsidies to ensure access to data plans. Providers sometimes adapted by changing delivery modalities to those requiring less bandwidth to accommodate those unable to afford good internet packages. Finding donated phones and computers for clients was frequently successful, but the cost of data and internet plans was a recurrent and ongoing problem.

*The issue with refugees, they have allowances from the federal government, it’s the resettlement assistance program and the allowance, so it’s not enough to pay for the Internet.*

(Mental health provider key informant 2)

#### 3.2.5. Appropriateness

Appropriateness refers to the quality of care, and its fit to client needs. In this study, we focused on the appropriateness of mental health care delivered virtually, rather than the appropriateness of mental health services as such.

Preferences for in-person care: Both clients and providers reported that virtual services were meeting client needs but many noted a preference for in-person care.

*[T]he hardest thing is you’re feeling… [exhales] You don’t feel yourself connected to the person you are talking to, as if you are watching tv [...].*

(Refugee newcomer 4)

Many clients reported that they were very satisfied with the mental health services they were receiving, but there was also a theme in many interviews of “making do” with the modality that was available, as in this quote below.

*I try to adapt to the situation, you know. But yeah, I don’t necessarily like it, but it’s not bad. It’s better than nothing.*

(Refugee newcomer 1)

Virtual modalities complicating communication: Several newcomers reported preferring to communicate directly with their mental health provider, without an interpreter, even if they did not understand all of what was said. But both newcomers and providers commented that communicating directly when you are not fluent in each other’s language can be even more challenging in virtual settings and that they did not always feel they could express themselves appropriately in some virtual modalities. Inhibited communication could further complicate cultural barriers to care. For example, phone conversations, in which non-verbal information is not available, could lead to misinterpretation, making interactions more difficult, as in the following quote.

*When it comes to a phone call, let us say now we may have language barrier and the way we talk as well; you may be talking with strong tone, which might seem that even if you are talking good words, they may feel you are yelling. [ehmmmm] this is because you have language problems you cannot express everything you want to say.*

(Refugee newcomer 5)

Privacy: Clients, providers, and interpreters frequently noted that a lack of privacy was an issue. Privacy could be an issue for providers and interpreters who were offering services from home and did not have appropriate office spaces from which to do so. For clients, crowding, interruptions, and thin walls were a challenge, with clients sometimes having their sessions in their cars, in parks, in libraries, or in coffee shops.

*Sometimes I would go outside. I was like “well, strangers, they will listen to me” but they don’t know me right?*

(Refugee newcomer 3)

Presumably those who were unable or uncomfortable being outside on their own would not have access to even this form of privacy.

Challenges to building a therapeutic relationship: For a variety of reasons, providers noted that it could be difficult to build therapeutic relationships online. Some providers noted that clients had distractions at home and were less engaged, which could make virtual service provision more challenging.

*[...] Yeah, I had clients run around the room or maybe sometimes you know it’s not attentive, surfing, browsing website while they talk to you or they use their phone or they talk to other people on social media during the meeting with you, that happened a lot.*

(Mental health provider 1)

In most cases, however, providers observed that VMH services just required different strategies, for instance, offering a first meeting in person before moving online, or adapting communication styles, reinforcing the need for training in virtual therapy techniques as well as technology.

*[B]ody language is missing quite a lot now and so generally it’s just upper body, so a lot more facial… I use my arms more so, to fill up this space, making a point. For example when a client’s crying… previously I might be able to pass a tissue box, and that is a message already. Meanwhile, now I have to compensate more verbally, [...] I might just have to fill up that he’s letting them know: “it’s OK, I’m still here, do you have a cup of water?” Do, you know, just whatever to fill up that space verbally.*

(Mental health provider key informant 4)

### 3.3. Dimensions of Service User’s Ability

#### 3.3.1. Ability to Perceive

The ability to perceive the need for care reflects potential clients’ knowledge and beliefs about health and sickness.

Stigma and lack of knowledge of symptoms/treatment: Both newcomers and providers noted that awareness that care is needed can be delayed or denied due to stigma in society or communities, or due to a lack of awareness of mental health symptoms and potential treatments. Friends, family members, or private sponsors were particularly important in helping to identify the need to seek care.

*… the wife comes complaining about the husband or my client has been referred by the sponsor because they saw an issue when they went, like how he’s treating his wife, how he’s behaving.*

(Settlement service provider 2)

Interestingly, many newcomers and providers felt that the growing awareness of stress during the COVID-19 pandemic may actually have made it easier for people in the community to reach out for care, as noted by this community leader.

*Distress was so much by COVID that they were going into a breaking down point, if it makes sense, and so some of them were actually kind of daring to kind of ask for help. COVID had made it possible for people to kind of seek help in a way because they were kind of breaking down that stigma; this is too much, I can’t manage it, the isolation and homeschooling and people was too much.*

(Community leader key informant 1)

#### 3.3.2. Ability to Seek Care

The ability to seek care is about clients’ autonomy and capacity to seek care. Issues of equity emerge here, as individuals or groups can face barriers to access care because of various structural or symbolic obstacles.

Feeling welcomed: Newcomers reported that feeling welcomed, as opposed to stigmatized, was an important part of accessing and continuing to access services; in the context of virtual care, they linked this to how they were treated in their initial (virtual) contacts.

*Even if you got the information to connect with them, since you are newcomer and settling in a new place, it may not be convenient to contact them. However, their welcoming attitude and support was important.*

(Refugee newcomer 6)

#### 3.3.3. Ability to Reach

The ability to reach focuses mostly on being physically able to reach services; we interpreted this as including physical access to the technology.

Inequality in technology access: Especially at the beginning of the pandemic, there were challenges accessing technology and the internet. These challenges were not distributed equally across the populations, they were common with higher poverty and lower education. Thus, some individuals and communities had better access to technology and devices and better digital literacy than others, and this difference tended to be gendered.

*I think for people who have difficulties are elderly, in navigating technology, and also for some women as well. The adult women, not the youth. [...] they may come from a village or they may never work before they came to Canada, so they just be home most of the time. So they’re not very computer literate as well. So those women [...] may have difficulties, and elderly.*

(Mental health provider 1)

As more community services moved online, however, the general comfort with and access to technology increased.

*Now there were very few clients who said: “I can’t do this, I have to stop therapy because I just don’t know how to do this online”. But our clients were actually more ingenious in adapting, [...] once we got used to Zoom, which we didn’t have very much time to get used to. Zoom, it’s quite comfortable, because there is space for the counselor, the interpreter, often other people join the client. You can see the whole family if you want to… It’s a little bit like a home visit and so things have worked surprisingly well. In fact, so well that when the time comes that we can go back to the in-person they probably still want to keep a mixture of in-person and online.*

(Agency director key informant 3)

Online schooling of children and online language classes facilitated digital literacy for the family as a whole. Newcomers also noted other community and peer supports were transitioning into virtual spaces as community members became more familiar with navigating physical distancing restrictions.

#### 3.3.4. Ability to Pay

The ability to pay refers to clients’ ability to access or generate the resources required to use the services.

The ongoing costs of virtual care: The cost of reliable internet or data plans was a pervasive issue for those refugee newcomers who struggled with low income, and it shaped which modalities of care were possible.

*I think I was lucky to be on O.W. [supplemental assistance for those with disabilities] and then I had a, like, a little bit of money to have, like, data on my phone, right? Otherwise, I know like some people...especially going to [name of organization removed], there are some people who are, like, struggling a lot with financial stuff and I wonder, like, how they get the services. And then if you, for example, if you prefer, like, a Zoom call, right, and you don’t have data or if you don’t have Wi-Fi, then how-- that’s challenging right?*

(Refugee newcomer 3)

This was balanced against managing the costs of transportation. Those refugee newcomers residing in areas that are far from services and who rely on public transportation face high costs for transportation, in time and money. The most vulnerable were those lacking the means for either technology or transportation.

#### 3.3.5. Ability to Engage

Ability to engage describes clients’ ability to participate in decision making regarding treatment. In the case of VMH, it could refer to making choices about the modality of treatment, type of interpretation services, and type of service itself, such as group versus individual therapy, as well as other aspects of treatment.

Feeling confident about engaging: Many newcomers stated that they actively engaged and made choices, but others described feeling there were no real choices and so not engaging:

*[...] she just asked me which one I would prefer, and I said whatever, since I would want to have in-person service. This might be the reason that she chose for me, I didn’t give her clear answer. *

(Refugee newcomer 7)

More could be done to ensure that clients are supported in knowing about their options and feeling able to engage in making choices, especially given that both clients and providers have stated that having choices is important.

## 4. Discussion

This study used Levesque et al.’s Client-Centered Framework to assess key stakeholders’ perceptions of access to VMH care for refugee newcomers, a vulnerable and underserved population. In line with Cu and colleagues’ scoping review [26], we found that the framework’s multiple dimensions could overlap; however, it provided a useful structure in the analysis, making visible the shared agency of providers and users in the process of accessing services. Our research also underlined the benefits of situating the model in a socioecological perspective, considering adaptations of providers and services to an unfolding health crisis.

This study on access to VMH care for refugee newcomers was conducted amid the public health restrictions of the COVID-19 pandemic. The transition to virtual services was abrupt, as most organizations were unprepared and unequipped to offer online services. Yet, providers and agencies were resourceful and committed to deliver care for their clients; all actors developed new knowledge, strategies, and preferences over the course of the pandemic, changing the prevalence and nature of barriers to care.

Byrow and colleagues’ [12] pre-pandemic review showed that refugees seeking mental health support encountered important barriers of three sorts: (1) structural (financial strain, language, housing, lack of information); (2) cultural (perceptions of health, mental health, and appropriate care; stigma); and (3) specific to refugee experience (immigration status, mistrust, preoccupation with confidentiality). With the move to virtual care during the pandemic, we found technology can reduce, exacerbate, or reconfigure these obstacles, particularly at the structural level, adding new challenges and opportunities.

Our analysis uncovered several themes related to the accessibility of VMH services and refugee clients’ abilities to access VMH care. First, virtual modalities offered the major advantage of allowing access to services across distances, removing the burdens of travel costs and time. They also created opportunities for access to services for refugee newcomers that might not otherwise be available, such as first-language therapists, also allowing connections for people living in rural or remote areas. However, the cost of technology and the complexity of virtual platforms and devices were a barrier for several clients. Interpreters themselves could face technological challenges. Internet service quality and fees as well as phone data plans were major concerns related to poverty. Connectivity issues, known to hinder access to VMH services [27,28], were frequently cited as a challenge in this study. For those in remote regions, where technology could bridge providers or interpreters who might not otherwise be available, such issues are a greater concern and may reduce the value of certain modalities in these regions. Respondents noted that refugee newcomers in Canada face elevated rates of poverty, and may have lower digital literacy and access to technology than other newcomers. The settlement agency ISSofBC [29] found that less than 40% of recently arrived refugees in British Columbia had a computer, and digital literacy was mostly limited to using WhatsApp. Greer and colleagues [30] found that the lack of digital literacy was the greatest barrier to using the internet for mental health services, highlighting the relevance of this barrier for refugee clients. Although additional resources were made available during the pandemic to support virtual services, they may be removed with the end of public health restrictions. Even as we transition to a growing use of technology, questions about sustainability thus remain.

The impact of technology on communication affected VMH appropriateness and access to care. Virtual care was often perceived as “making do” by refugee newcomers and providers, as in-person services were limited by the pandemic. Participants mentioned the ways in which various modalities affected the communication quality and challenged the creation of a trusting relationship and alliance, a major component of care. Indeed, the relationship between therapist and client is one of the most important factors in therapeutic efficacy [31,32]. When working with refugees and other displaced populations, therapists already must contend with language barriers, interpreters [33], cultural differences [34,35], and lack of familiarity/comfort with Eurocentric-based models when building the therapeutic relationship [36]. These challenges may have been exacerbated by VMH, hindering the connection between service providers and service users in specific ways [37]. Providers however developed strategies to accommodate the virtual modality, including offering initial in-person sessions or by compensating with gestures or more words. Issues of comfort, privacy, and safety when using VMH services were also discussed as impacting access in important ways. Ensuring safe and private physical environments for care was a major challenge, often linked to poverty, crowding, and/or poor-quality housing—known barriers to VMH services [30]. Our results also echo a study of online mental health consultations in 13 European countries during the pandemic, showing that the concern most cited by participants was “privacy and security” [27]. On a related note, however, given that mental health stigma also appears to be an important barrier to mental health care for refugees [38], and VMH could provide a more discreet way to have access, if provided services are perceived as safe. Achieving perceptions of safety may be challenging though; forced migrants’ specific experiences often involve violence, persecution, and trauma, leading some individuals to develop profound difficulties with trust and worries about confidentiality (risks for oneself and relatives if trust is broken) [39], affecting the capacity to engage in care [40]. In our study, some found building trust in virtual platforms with interpreters or providers was an additional challenge.

Considering all previously mentioned themes and dimensions, perhaps the most important and overarching theme concerns flexibility, as a key element favoring access. While the population had very little choice regarding confinement measures, our findings suggest that providing a choice of different modalities to newcomers (including for in-person services) could improve access in different situations, for different kinds of services, for different mental health concerns, and different individuals. Given the heterogeneity of refugee newcomers’ experiences and needs, offering alternatives, and ensuring clients are supported in making choices, including for in-person care, appears to be an important factor to reduce each person’s singular set of obstacles to mental health services. There is still limited research on refugee clients’ ability to exercise their rights in choosing their service modality however, including in contexts where choice would be limited and affected by a global crisis such as the pandemic [41]. Finally, our findings underline the importance of support and training for providers who refer and who offer mental health services.

### 4.1. Limitations and Future Research

A few considerations should be taken into account when examining our findings. We collected the perspectives of refugee newcomers who could and did use virtual modalities, and who had experience with the mental health care system (most likely not for severe mental health problems). Those who found VMH care unacceptable or inaccessible would not have been able to participate in the study, both because of our inclusion criteria, but also because we used virtual technologies to conduct the interviews. Moreover, although we recruited from different provinces, this article did not explore comparisons between sites in terms of pandemic health measures and coverage of mental health services for refugees. Finally, we documented perceptions regarding any VMH care, although this term encompasses very different services and modalities. Yet, our research highlights important and still unexplored issues regarding access to (virtual) mental health care for a vulnerable and underserved population during the pandemic. Future studies could examine the perceptions of a wider range of newcomers, including those unable to use virtual modalities, or go more deeply into unique barriers experienced by individuals suffering from severe mental health problems, women, seniors, and people identifying as LGBTQ.

### 4.2. Conclusions

As we transition back to more in-person care, almost all providers noted a desire to retain some elements of VMH care in the future. Thus, although the circumstances of delivering mental health services during the pandemic were unique, they also offered opportunities to learn more about whether, for whom, when, and how virtual mental health care increases access to services. VMH services rapidly expanded in the COVID-19 context and have the potential to bridge gaps between refugee mental health care needs and available services. However, most virtual health initiatives are not sustained because of a lack of research on user needs, goals, and perceptions [17,18], and fail to address accessibility barriers for disadvantaged patients [42]. This study identified a number of characteristics of VMH services that could interact with clients’ abilities, to either limit or enhance access to needed mental health care. Importantly, virtual modalities differed in accessibility as a function of the services offered, client needs, abilities, and preferences, and the resources available, reinforcing the importance of flexibility and choice in VMH services to reflect the diversity of refugee client circumstances and needs.

## Figures and Tables

**Table 1:** Distribution of Service Providers’ Interviews by Province and Role.

Key Informant Service Provider Interviews (*n* = 32)					
	AB ^1^	BC ^2^	ON ^3^	QC ^4^	Total
Program coordinators	0	0	1	2	3 (9.4%)
Managers and directors	0	2	3	1	6 (18.8%)
Settlement workers	0	0	1	4	5 (15.6%)
Primary care providers	0	1	0	0	1 (3.1%)
Mental health providers	1	2	7	4	14 (43.8%)
Interpreters	0	0	2	1	3 (9.4%)
Follow-up Service Provider Interviews (*n* = 31)					
Program coordinators/ intake workers	2	2	0	0	4 (12.9%)
Settlement workers	0	0	4	6	10 (32.2%)
Primary care providers	1	0	2	3	6 (19.4%)
Mental health providers	0	3	5	3	11 (35.5%)

^1^ AB = Alberta. ^2^ BC = British Columbia. ^3^ ON = Ontario. ^4^ QC = Québec.

**Table 2:** Sociodemographic Information of Refugee Newcomer Clients (*n* = 37).

Refugee Newcomer Clients (*n* = 37)		
Province of residence	*n*	%
AB	7	18.9
BC	5	13.5
ON	22	59.5
QC	3	8.1
Country of origin		
Syria	7	18.9
Eritrea	7	18.9
Iran	5	13.5
Ethiopia	4	10.8
Columbia	3	8.1
Somalia	2	5.4
Other	9	24.3
Years residing in Canada		
<1	4	10.8
1–2	20	54.0
2–3	6	16.2
4–5	7	18.9
First language		
Arabic	7	18.9
Spanish	7	18.9
Farsi	6	16.2
Tigrinya	6	16.2
Amharic	5	13.5
Somali	2	5.4
Other	4	10.8

## Data Availability

Data available on request due to restrictions. The data presented in this study are available on request from the corresponding author. The data are not publicly available due to privacy concerns of qualitative data.
